# Supplementary material for: Etrolizumab-s fails to control E-Cadherin-dependent co-stimulation of highly activated cytotoxic T cells
Source: Nat Commun. 2024 Feb 3;15:1043. doi: 10.1038/s41467-024-45352-6 (PMC10838339; doi:10.1038/s41467-024-45352-6)
Supplement: Supplementary file 1 — Supplementary Information [file 41467_2024_45352_MOESM1_ESM.pdf]

Etrolizumab-s fails to control E-Cadherin-dependent co-stimulation of highly activated cytotoxic T cells

Maximilian Wiendl[1], Mark Dedden[1], Li-Juan Liu[1], Anna Schweda[1], Eva-Maria Paap[1], Karen A-M. Ullrich[1], Leonie Hartmann[1], Luisa Wieser[1], Francesco Vitali[1], Imke Atreya[1,2], Tanja M. Müller[1,2], Claudia Günther[1,2], Raja Atreya[1,2], Markus F. Neurath[1,2], Sebastian Zundler[1,2,*]

[1] Department of Medicine 1, University Hospital Erlangen and Friedrich-Alexander-Universität Erlangen-Nürnberg, Germany

[2] Deutsches Zentrum Immuntherapie, University Hospital Erlangen, Germany

Supplementary Information:

Supplementary Figures 1-8

Supplementary Tables 1-2

Supplementary Figure 1

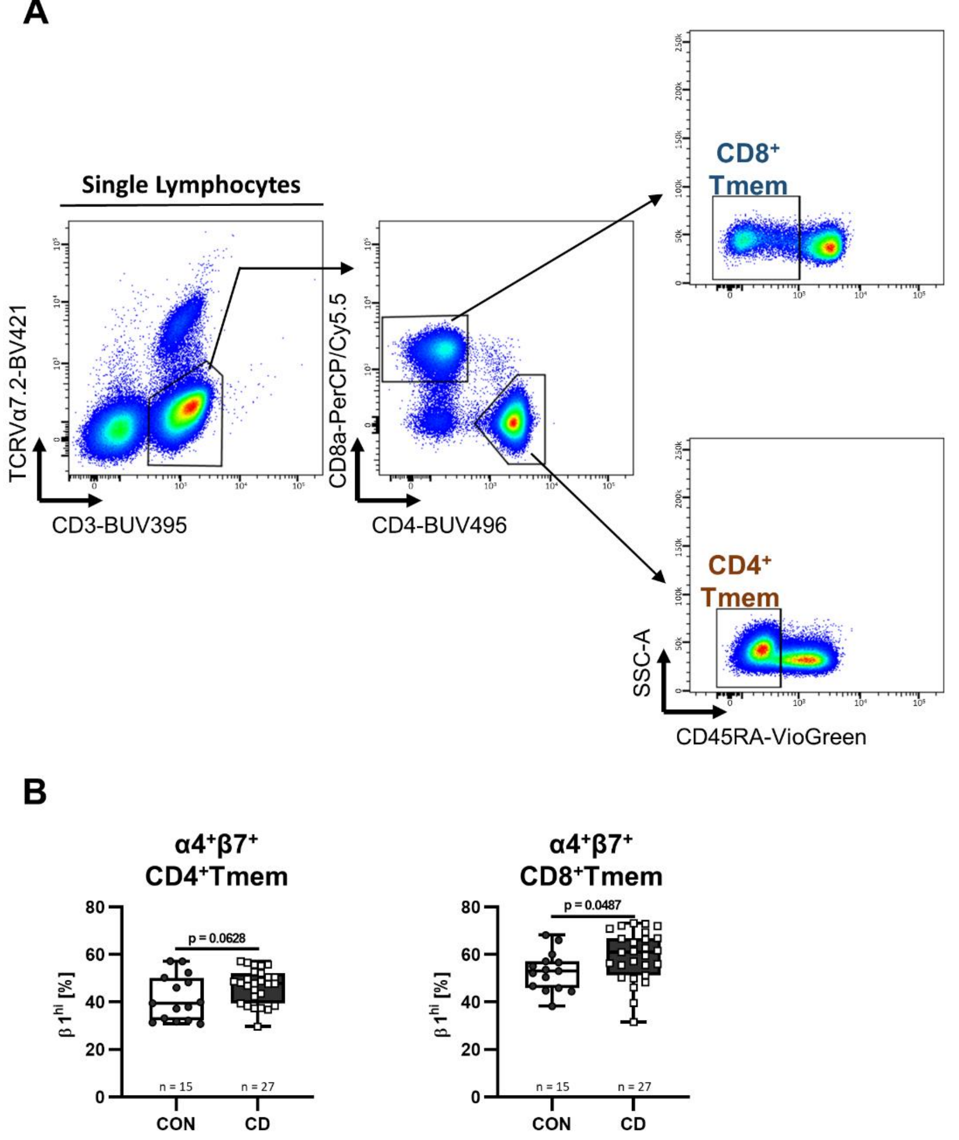

Supplementary Figure 1:

(A) Representative gating of peripheral blood $CD3^{+}TCRVa7.2^{-}CD4^{+/-}CD8a^{+/-}CD45RA^{-/lo/neg}$ $CD4^{+}$ and $CD8^{+}$ memory T cells (Tmem) in flow cytometry analysis. After doublet exclusion on living lymphocytes (as determined by FSC/SSC), we selected for $CD3^{+}TCRVa7.2^{-}$ followed by the selection of either $CD4^{+}CD8a^{-}$ or $CD4^{-}CD8a^{+}$ T cells. Subsequently $CD4^{+}$ Tmem and $CD8^{+}$ Tmem were selected as $CD45RA^{neg}$ and $CD45RA^{lo/neg}$, respectively. **(B)** Quantitative flow cytometry of β1 expression on $α4^{+}β7^{+}$ $CD4^{+}$ (left) and $CD8^{+}$ (right) Tmem from non-IBD controls (CON, n = 15) and patients with Crohn's disease (CD, n = 27).

Significant outliers were detected by 1% ROUT test and removed from analysis. Normality was determined by D'Agostino and Pearson test and two-tailed unpaired t or Mann-Whitney tests were performed accordingly. Data are displayed as box-whisker plots from minimum to maximum. Source data are provided as a Source Data file.

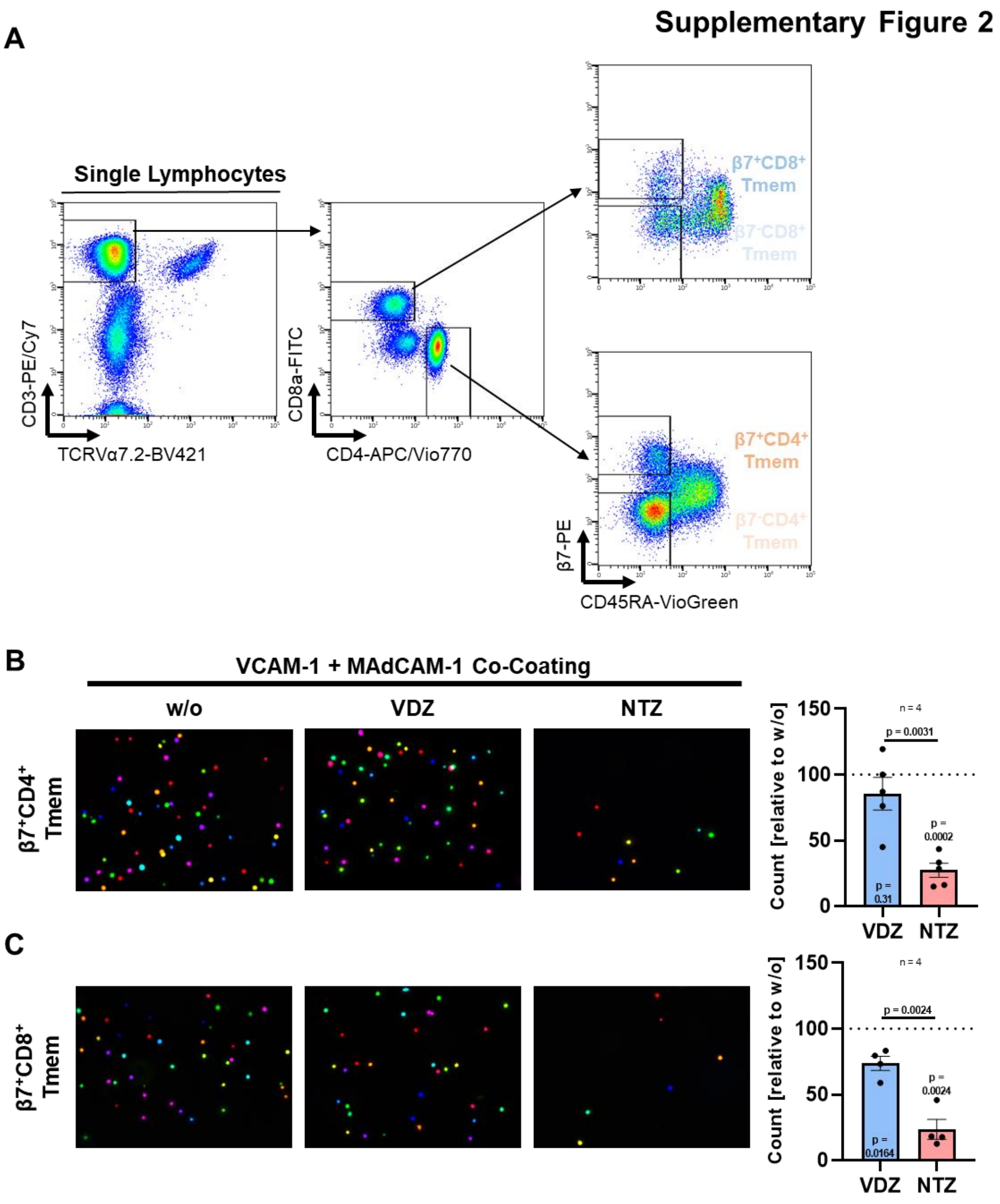

Supplementary Figure 2:

(A) Representative gating of peripheral blood CD3^{+}TCRVa7.2^{-}CD4$^{+/-}$CD8a$^{+/-}$CD45RA^{-} b7$^{+/-}$ Tmem for fluorescence-activated cell sorting. After doublet exclusion on living lymphocytes (as determined by FSC/SSC), we selected for CD3^{+}TCRVa7.2^{-} followed by the selection of either CD4^{+}CD8a^{-} or CD4^{-}CD8a^{+} T cells. Subsequently Tmem were determined as CD45RA^{-} and sorted according to β7 expression. **(B,C)** Representative stacks of all twelve microscopic images acquired per capillary (left) and relative

quantification (right) of dynamic adhesion assays with sorted β7^{+} CD4^{+} Tmem (**B**, CD3^{+}TCRVα7.2^{-}CD4^{+}CD8a^{-}CD45RA^{-}β7^{+}) or β7^{+} CD8^{+} Tmem (**C**, CD3^{+}TCRVα7.2^{-}CD4^{-}CD8a^{+}CD45RA^{-}β7^{+}) on glass capillaries co-coated with MAdCAM-1 and VCAM-1 after incubation with or without 10 µg/ml vedolizumab (VDZ) or the anti-α4 antibody natalizumab (NTZ). n = 4-5 per condition.

Significant outliers were detected by 1% ROUT test and removed from analysis. Normality was determined by Shapiro-Wilk test and two-tailed paired t and one sample t tests or two-tailed Wilcoxon matched-pairs signed rank and one sample Wilcoxon signed rank tests were performed accordingly. Data are displayed as bar plots depicting mean +/- SEM. Source data are provided as a Source Data file.

Supplementary Figure 3

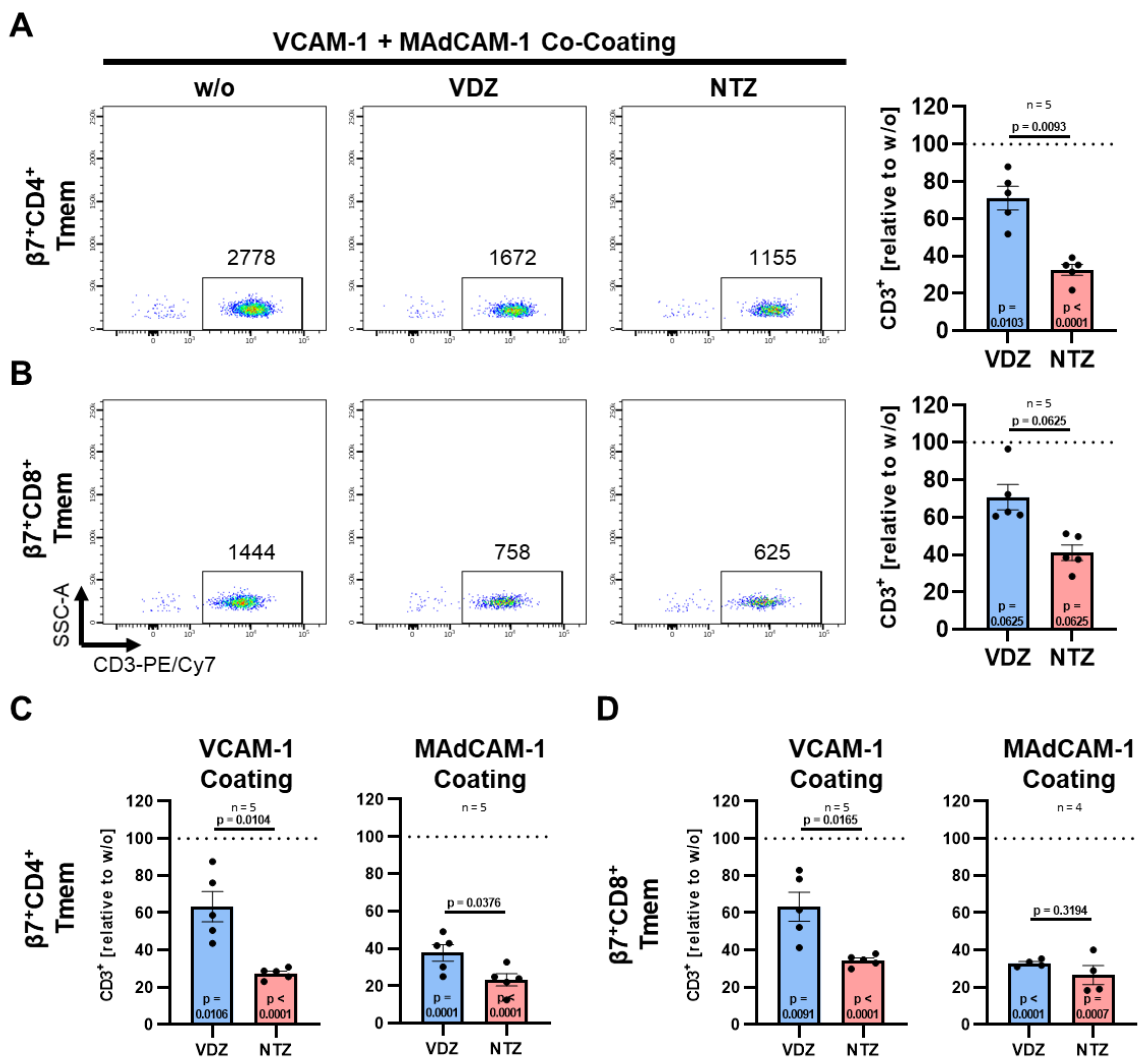

Supplementary Figure 3

(A, B) Representative (left) and quantitative flow cytometry (right) of transmigration assays with sorted β7^+ CD4^+ (**A**) or β7^+ CD8^+ (**B**) Tmem after migration over porous membranes co-coated with MAdCAM-1 and VCAM-1 after incubation with or without 10 µg/ml VDZ or the anti-α4 antibody NTZ. n = 5.

(C) Relative flow cytometry of sorted β7^+CD4^+ Tmem (CD3^+TCRVα7.2^-CD4^+CD8a^-CD45RA^-β7^+) transmigrated over membranes coated with MAdCAM-1 (left) or VCAM-1 (right) after incubation with or without 10 µg/ml VDZ or the anti-α4 antibody NTZ. n = 5. **(D)** Relative flow cytometry of sorted β7^+CD8^+ Tmem (CD3^+TCRVα7.2^-CD4-CD8a^+CD45RA^-β7^+) transmigrated over membranes coated with MAdCAM-1 (left) or

VCAM-1 (right) after incubation with or without 10 µg/ml VDZ or the anti-α4 antibody NTZ. n = 4-5.

Significant outliers were detected by 1% ROUT test and removed from analysis. Normality was determined by Shapiro-Wilk test and two-tailed paired t and one sample t tests or two-tailed Wilcoxon matched-pairs signed rank and one sample Wilcoxon signed rank tests were performed accordingly. Data are displayed as bar plots depicting mean +/- SEM. Source data are provided as a Source Data file.

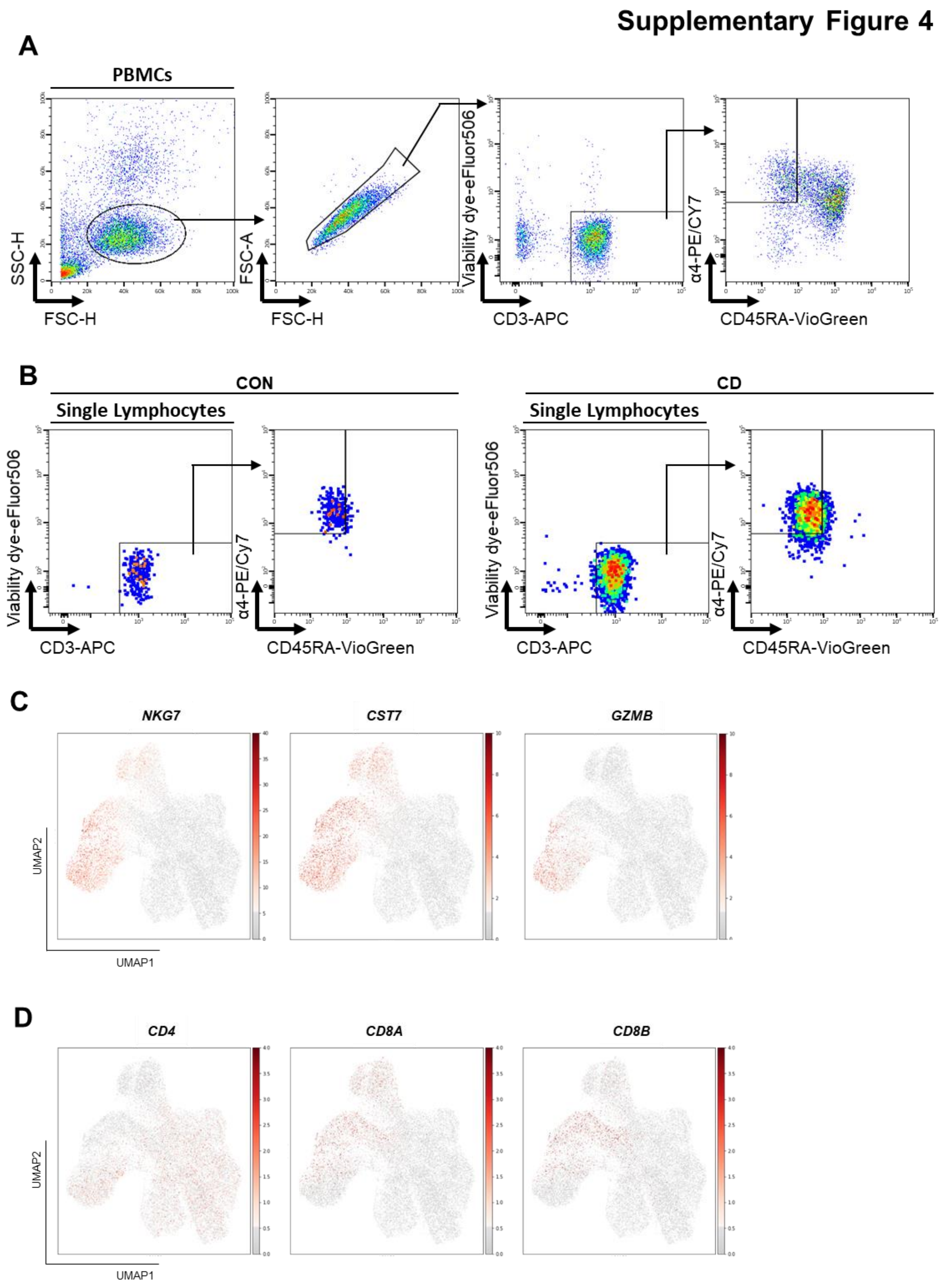

Supplementary Figure 4:

(A) Representative gating of viable peripheral blood $CD3^{+}CD45RA^{-}\alpha4^{+}$ Tmem for sorting. After doublet exclusion on lymphocytes (as determined by FSC/SSC), viable

T cells were selected by gating on CD3$^+$Viability dye$^-$ cells. Next, α4$^+$CD45RA$^-$ cells were selected. **(B)** Purity controls from the sort of the healthy control (CON) and the Crohn's disease (CD) patient for single cell RNA sequencing. **(C,D)** UMAP plots showing the distribution of cells expressing indicated genes. Source data are provided as a Source Data file.

Supplementary Figure 5

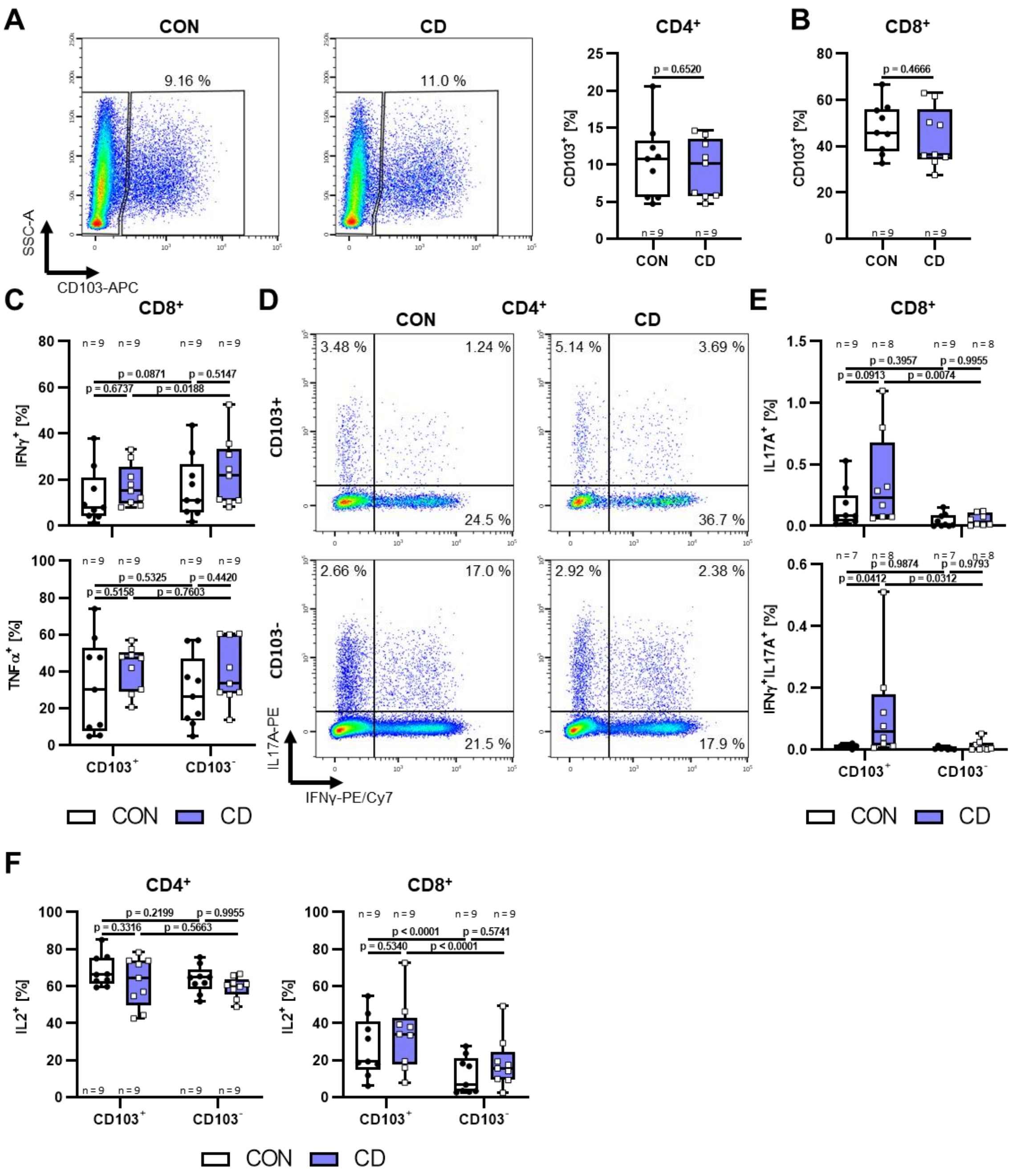

Supplementary Figure 5:

(A,B) Representative and quantitative flow cytometry of CD103 expression on magnetically enriched peripheral CD4$^+$ **(A)** and CD8$^+$ (**B)** T cells after 3 days of stimulation with anti-CD2/3/28 beads in the presence of 10 ng/ml TGFβ1, overnight resting and subsequent 4 h of restimulation with PMA/Ionomycin and Brefeldin A/Monensin. Significant outliers were detected by 1% ROUT test and removed from

analysis. Normality was determined by D'Agostino and Pearson test and two-tailed unpaired t tests were performed accordingly. Data are displayed as box-whisker plots from minimum to maximum. **(C,E)** Quantitative flow cytometry of IFNγ, TNFα, IL17A and IL17A/IFNγ co-expression by $CD103^{+/-}$ $CD8^{+}$ T cells cultured according to **(A). (D)** Representative flow cytometry of IL17A and IFNγ expression on $CD103^{+/-}$ $CD4^{+}$ T cells cultured according to **(A). (F)** Quantitative flow cytometry of IL2 expression on $CD103^{+/-}$ $CD4^{+}$ (left) and $CD8^{+}$ (right) T cells cultured according to **(A).** Significant outliers were detected by 1% ROUT test and removed from analysis. Two-way ANOVAs with Sidak's multiple comparisons test were performed. Data are displayed as box-whisker plots from minimum to maximum.

n = 7 - 9 patients with CD or non-IBD controls (CON) as indicated.

Source data are provided as a Source Data file.

Supplementary Figure 6

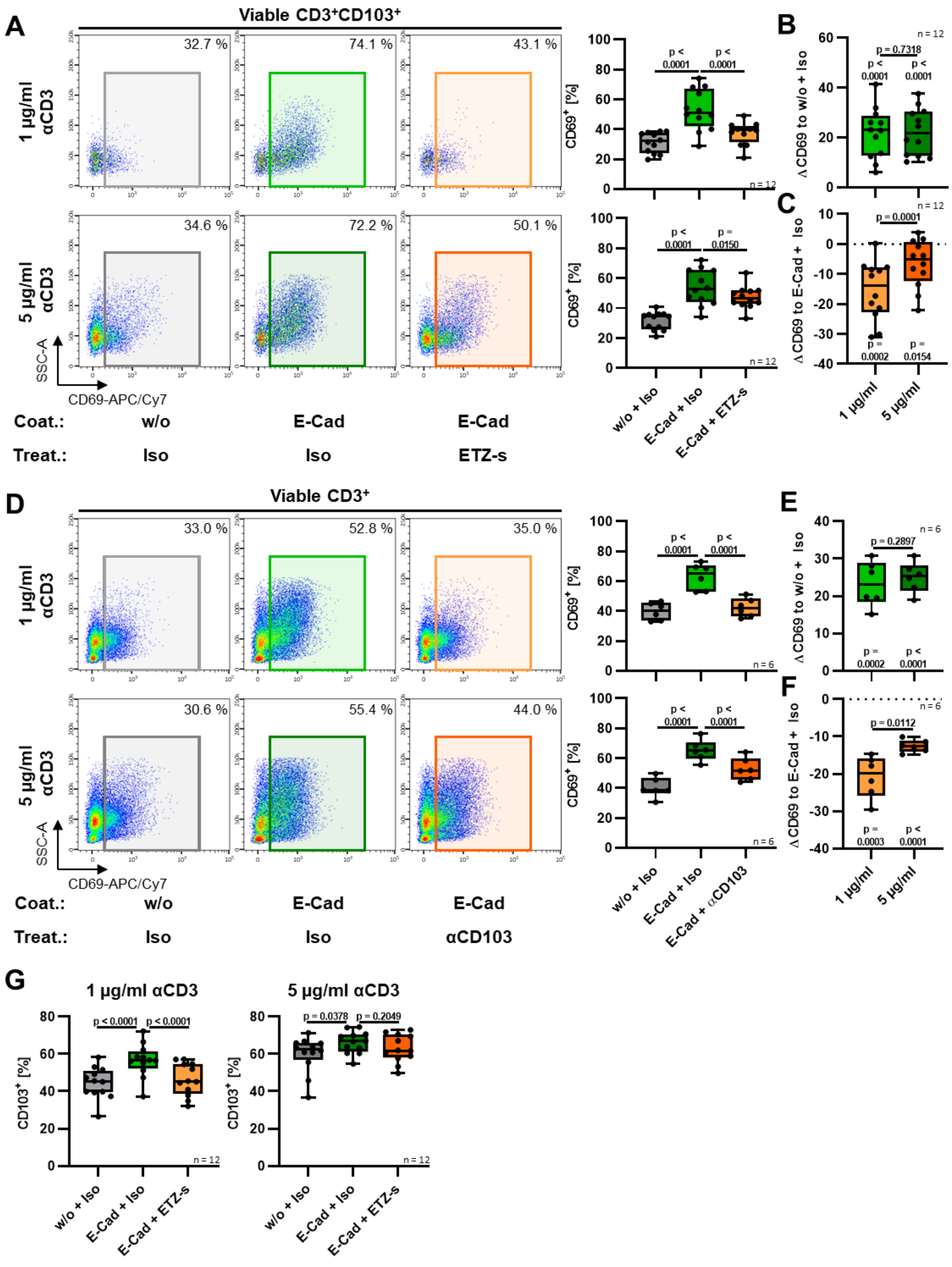

Supplementary Figure 6:

(A-C) Representative and quantitative flow cytometry of CD69 expression on induced $CD8^{+}CD103^{+}$ peripheral blood T cells after re-stimulation on plates coated with either

1 or 5 µg/ml anti-CD3 together with or without (w/o) E-Cadherin (E-Cad) in the presence of 10 µg/ml ETZ-s or corresponding isotype control (Iso). Significant outliers were detected by 1% ROUT test and removed from analysis. Normality was determined by D'Agostino and Pearson test RM one-way ANOVAs with Dunnett's multiple comparisons test was performed accordingly.**(B)** Change of CD69 expression in reference to w/o + Iso. **(C)** Change of CD69 expression in reference to E-Cad + Iso. Significant outliers were detected by 1% ROUT test and removed from analysis. Normality was determined by D'Agostino and Pearson test and two-tailed paired t and one sample t tests were performed accordingly. n = 12. **(D-F)** Representative and quantitative flow cytometry of CD69 expression on induced $CD8^{+}$CD103+ peripheral blood T cells re-stimulated in the same way and treated with 10 µg/ml anti-CD103 or corresponding isotype control (Iso). Significant outliers were detected by 1% ROUT test and removed from analysis. Normality was determined by D'Agostino and Pearson test RM one-way ANOVAs with Dunnett's multiple comparisons test was performed accordingly. **(E)** Change of CD69 expression in reference to w/o + Iso. **(F)** Change of CD69 expression in reference to E-Cad + Iso. Significant outliers were detected by 1% ROUT test and removed from analysis. Normality was determined by Shapiro-Wilk test and two-tailed paired t and one sample t tests were performed accordingly. n = 6. **(G)** Quantitative flow cytometry of CD103 expression on induced $CD8^{+}$ peripheral blood T cells after re-stimulation as detailed above in the presence of 10 µg/ml ETZ-s or corresponding isotype control (Iso). Significant outliers were detected by 1% ROUT test and removed from analysis. Normality was determined by D'Agostino and Pearson test RM one-way ANOVAs with Dunnett's multiple comparisons test was performed accordingly.

Data are displayed as box-whisker plots from minimum to maximum. Source data are provided as a Source Data file.

Supplementary Figure 7

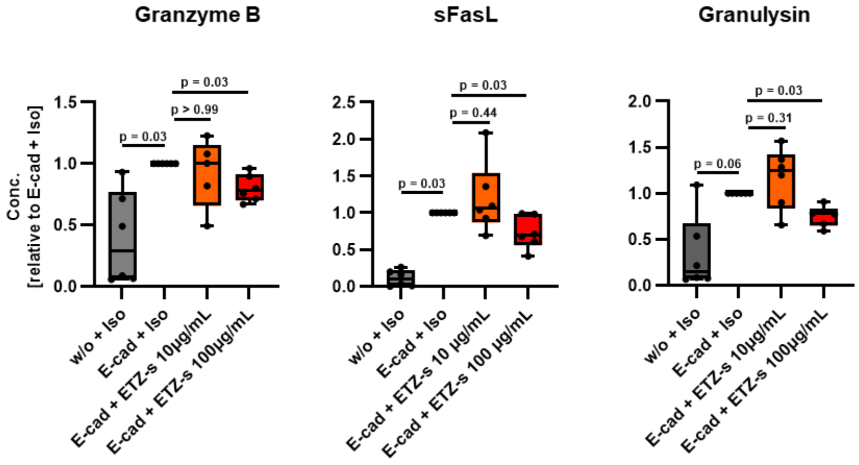

Supplementary Figure 7: Relative concentrations of the indicated proteins in the supernatants of re-stimulated cultures relative to E-Cad + Iso. Significant outliers were detected by 1% ROUT test and removed from analysis. Two-tailed one sample Wilcoxon signed rank tests were performed. n = 6.

Data are displayed as box-whisker plots from minimum to maximum. Source data are provided as a Source Data file.

Supplementary Figure 8

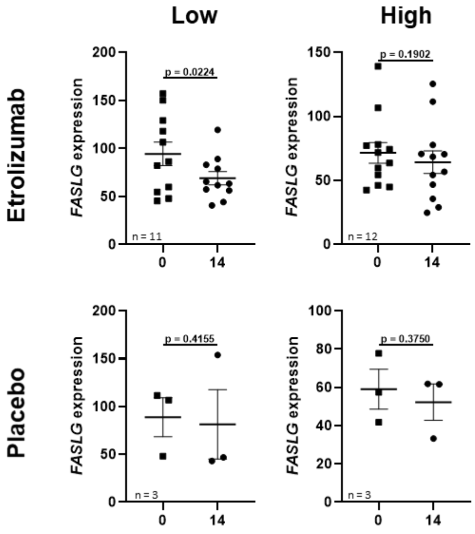

Supplementary Figure 8:

Expression of the indicated genes in RNA sequencing data from ileal samples of the BERGAMOT cohort 1 according to low or high T cell activation scores and treatment with etrolizumab or placebo. Significant outliers were detected by 1% ROUT test and removed from analysis. Normality was determined by D'Agostino and Pearson (n>=10) or Shapiro-Wilk test (n<10) test and one-tailed paired t or one-tailed Wilcoxon matched pairs signed rank tests were performed accordingly. Data are displayed as dot plots with mean +/- SEM. Source data are provided as a Source Data file.

Supplementary Table 1:

Characteristics of blood donors

		CON	CD
**Number donors**		31	45
**Number samples**		69	50
**Age (Ø, range)**		27.9, 22-45	40.6, 22-72
**Female [%]**		67.7	48.9
**HBI (Ø, range)**			3.7, 0-16
**Therapy [%}**	**Azathioprine**		4.4
	**5-ASA**		6.6
	**Methotrexate**		4.4
	**Steroids**		15.6
	**Anti-TNF**		86.7
	**Ustekinumab**		13.3
**Location [%]**	**L1**		26.7 (+4: 6.7)
	**L2**		15.6 (+4: 0)
	**L3**		57.8 (+4: 17.8)

Supplementary Table 2:

Characteristics of blood donors treated with vedolizumab

	UC	CD
**Number donors**	41	20
**Age (Ø, range)**	45.2, 20-79	37.5, 20-59
**Female [%]**	46.3	70.0
**HBI (Ø, range)**		7.9, 3-15
**PMS (Ø, range)**	3.8, 1-9	
**Location [%]**	E1: 17.1	L1: 20.0
	E2: 46.3	L2: 15.0
	E3: 36.6	L3: 65.0
