# Supplementary material for: Etrolizumab-s fails to control E-Cadherin-dependent co-stimulation of highly activated cytotoxic T cells
Source: Nat Commun. 2024 Feb 3;15:1043. doi: 10.1038/s41467-024-45352-6 (PMC10838339; doi:10.1038/s41467-024-45352-6)
Supplement: Supplementary file 3 — Reporting Summary [file 41467_2024_45352_MOESM3_ESM.pdf]

nature portfolio

Corresponding author(s): Sebastian Zundler

Last updated by author(s): Dec 28, 2023

Reporting Summary

Nature Portfolio wishes to improve the reproducibility of the work that we publish. This form provides structure for consistency and transparency in reporting. For further information on Nature Portfolio policies, see our Editorial Policies and the Editorial Policy Checklist.

Statistics

For all statistical analyses, confirm that the following items are present in the figure legend, table legend, main text, or Methods section.

n/a	Confirmed	
☐	☒	The exact sample size (n) for each experimental group/condition, given as a discrete number and unit of measurement
☐	☒	A statement on whether measurements were taken from distinct samples or whether the same sample was measured repeatedly
☐	☒	The statistical test(s) used AND whether they are one- or two-sided<br>*Only common tests should be described solely by name; describe more complex techniques in the Methods section.*
☒	☐	A description of all covariates tested
☐	☒	A description of any assumptions or corrections, such as tests of normality and adjustment for multiple comparisons
☐	☒	A full description of the statistical parameters including central tendency (e.g. means) or other basic estimates (e.g. regression coefficient) AND variation (e.g. standard deviation) or associated estimates of uncertainty (e.g. confidence intervals)
☐	☒	For null hypothesis testing, the test statistic (e.g. F, t, r) with confidence intervals, effect sizes, degrees of freedom and P value noted<br>*Give P values as exact values whenever suitable.*
☒	☐	For Bayesian analysis, information on the choice of priors and Markov chain Monte Carlo settings
☒	☐	For hierarchical and complex designs, identification of the appropriate level for tests and full reporting of outcomes
☒	☐	Estimates of effect sizes (e.g. Cohen's d, Pearson's r), indicating how they were calculated

Our web collection on statistics for biologists contains articles on many of the points above.

Software and code

Policy information about availability of computer code

Data collection	RNA sequencing: 10X Chromium Controller<br>Flow cytometry: LSR Fortessa (BD Bioscience), MACS Quant 10, MACS Quant 16 (Miltenyi)
Data analysis	RNA sequencing: 10x CellRanger v7.0.0, scanpy v1.7.2, DeSeq2 v1.24.0, R v3.6.1,<br>FlowJo v10.7.1 (Tree Star)

For manuscripts utilizing custom algorithms or software that are central to the research but not yet described in published literature, software must be made available to editors and reviewers. We strongly encourage code deposition in a community repository (e.g. GitHub). See the Nature Portfolio guidelines for submitting code & software for further information.

Data

Policy information about availability of data

All manuscripts must include a data availability statement. This statement should provide the following information, where applicable:

- Accession codes, unique identifiers, or web links for publicly available datasets
- A description of any restrictions on data availability
- For clinical datasets or third party data, please ensure that the statement adheres to our policy

Single cell transcriptomic data generated in this study have been deposited in the GEO database under the accession code GSE252122 [https://

www.ncbi.nlm.nih.gov/geo/query/acc.cgi?acc=GSE252122]. The flow cytometry and adhesion assay data that support the findings of this study are available from the corresponding author upon reasonable request. Source data are provided with this paper.

Research involving human participants, their data, or biological material

Policy information about studies with human participants or human data. See also policy information about sex, gender (identity/presentation), and sexual orientation and race, ethnicity and racism.

Reporting on sex and gender	Sex was determined based on self-reporting. 68 % of the control patients and 49 % of the patients with CD were female. The results show aggregated data from all donors. Sex was not considered in the study design and consent has not been obtained for sharing individual-level data, since sex-specific differences in the aspects investigated were not suspected.
Reporting on race, ethnicity, or other socially relevant groupings	None.
Population characteristics	Population characteristics are summarized in Suppl. Table 1.
Recruitment	Patients presenting at the Outpatient and Endoscopy Department of the Department of Medicine 1 of the University Hospital Erlangen as well as non-IBD controls were asked for participation by the treating physician in the study after informed consent. We do not assume that there was a relevant self-selection bias, since virtually all patients approached consented to take part.
Ethics oversight	Ethics Committe of the Friedrich-Alexander-Universität Erlangen-Nürnberg.

Note that full information on the approval of the study protocol must also be provided in the manuscript.

Field-specific reporting

Please select the one below that is the best fit for your research. If you are not sure, read the appropriate sections before making your selection.

☒ Life sciences ☐ Behavioural & social sciences ☐ Ecological, evolutionary & environmental sciences

For a reference copy of the document with all sections, see nature.com/documents/nr-reporting-summary-flat.pdf

Life sciences study design

All studies must disclose on these points even when the disclosure is negative.

Sample size	Sample sizes were determined based on effects observed in previous studies where applicable or based on the availability of donors and the analysis capacity.
Data exclusions	1% ROUT tests were performed to dectect significant outliers. These outliers where then excluded from the analysis.
Replication	Each experiment was performed at least two times independently and all attempts at replication were successful.
Randomization	Since it was not an interventional study, there was no randomization performed.
Blinding	For all in vitro experiments, the investigators were blinded to the group allocations during the measurements and data analysis. The blinding was not possible in obtaining PBMCs and in in silico analyses of transcriptomic datasets.

Reporting for specific materials, systems and methods

We require information from authors about some types of materials, experimental systems and methods used in many studies. Here, indicate whether each material, system or method listed is relevant to your study. If you are not sure if a list item applies to your research, read the appropriate section before selecting a response.

Materials & experimental systems

n/a	Involved in the study	
☐	☒	Antibodies
☒	☐	Eukaryotic cell lines
☒	☐	Palaeontology and archaeology
☒	☐	Animals and other organisms
☐	☒	Clinical data
☒	☐	Dual use research of concern
☒	☐	Plants

Methods

n/a	Involved in the study	
☒	☐	ChIP-seq
☐	☒	Flow cytometry
☒	☐	MRI-based neuroimaging

Antibodies

Antibodies used

Antibodies for in vitro treatments:
vedolizumab (Takeda), natalizumab (Biogen), ETZ-s (BioXcell [#BE0062]), IgG from rat serum (Merck [I4131]), CD103 (Biolegend [350202]), Purified Mouse IgG1, κ Isotype Ctrl Antibody [400102]).
Flow cytometry:
CD3 (BUV395, SK7, BD Biosciences [564001] / APC, HIT3a, Biolegend [300312] / FITC, OKT3, Biolegend [317306], PE/Cy7, SK7, Biolegend [344816]), CD4 (BUV496, SK3, BD Biosciences [612937] / VioBlue /APC-Vio770, VIT4, Miltenyi Biotec [130-113-781/130-113-211]), CD8a (PerCP/Cy5.5 / FITC, RPA-T8, Biolegend [301032/301006]), CD25 (FITC, M-A251, Biolegend [356106]), CD45RA (VioGreen, REA1047, Miltenyi Biotec [130-117-744]/ APC/Cy7, HI100, Biolegend [304128]), CD69 (APC/Cy7, FN50, Biolegend [310914]), CD103 (APC / PE/Cy7, Ber-ACT8, Biolegend [350216/350212]), CCR9 (PE/Cy7, L053E8, Biolegend [358910]), TCRVα7.2 (BV421, OF5A12, BD Biosciences [749494]), α4 (FITC, MZ18-24A9, Miltenyi Biotec [130-122-933] / PE/Cy7, 9F10, Biolegend [304313]), β1 (AF488 / PE, TS2/16, Biolegend [303003/303016]), β7 (BV605, FIB504, BD Biosciences [564284] / PE, FIB27, Biolegend [121006]), GNLY (PE, DH2, Biolegend [348004]), GZMB (PacificBlue, GB11, Biolegend [515408]), GZMK (APC, GM26E7, Biolegend [370510]), IFNγ (PE/Cy7, B27, Biolegend [506528]), IL2 (BV421, 5344.111, BD Biosciences [562914]), IL17A (PE, BL168, Biolegend [512306]), TNFα (PE, Mab11, Biolegend [502909])

Validation

All commercially obtained antibodies were previously validated by the manufacturer for the specific application. The validation statements can be retrieved on the manufacturers' webpages by searching for the indicated reference number.

Clinical data

Policy information about clinical studies
All manuscripts should comply with the ICMJE guidelines for publication of clinical research and a completed CONSORT checklist must be included with all submissions.

Clinical trial registration: n.a.

Study protocol: n.a.

Data collection: n.a.

Outcomes: n.a.

Plants

Seed stocks: n.a.

Novel plant genotypes: n.a.

Authentication: n.a.

Flow Cytometry

Plots

Confirm that:

☒ The axis labels state the marker and fluorochrome used (e.g. CD4-FITC).

☒ The axis scales are clearly visible. Include numbers along axes only for bottom left plot of group (a 'group' is an analysis of identical markers).

☒ All plots are contour plots with outliers or pseudocolor plots.

☒ A numerical value for number of cells or percentage (with statistics) is provided.

Methodology

Sample preparation	Single cell preparations from peripheral blood were individually prepared as described in the Methods section.
Instrument	Collection: MACSQuant10 and 16 (Miltenyi) or LSR Fortessa (BD Bioscience)<br>Analysis: FlowJo v10.8.1 (Treestar)
Software	Collection: MACSQuant10 and 16 (Miltenyi) or LSR Fortessa (BD Bioscience)<br>Analysis: FlowJo v10.7.1 (Treestar)
Cell population abundance	Purity of cell populations isolated by FACS or MACS was typically > 95 %.
Gating strategy	Gating strategies are described in the main text and/or figure legends.

☒ Tick this box to confirm that a figure exemplifying the gating strategy is provided in the Supplementary Information.
